# Preferences on Governance Models for Mental Health Data: Qualitative Study With Young People

**DOI:** 10.2196/50368

**Published:** 2024-04-23

**Authors:** Emma Grace Carey, Faith Oluwasemilore Adeyemi, Lakshmi Neelakantan, Blossom Fernandes, Mina Fazel, Tamsin Ford, The MindKind Consortium, Anne-Marie Burn

**Affiliations:** 1 Department of Psychiatry University of Cambridge Cambridge United Kingdom; 2 Department of Psychology University of Bath Bath United Kingdom; 3 School of Population and Global Health University of Melbourne Melbourne Australia; 4 Department of Psychiatry University of Oxford Oxford United Kingdom; 5 See Acknowledgments

**Keywords:** young people, mental health, data, governance, deliberative democracy, mobile phone

## Abstract

**Background:**

Improving access to mental health data to accelerate research and improve mental health outcomes is a potentially achievable goal given the substantial data that can now be collected from mobile devices. Smartphones can provide a useful mechanism for collecting mental health data from young people, especially as their use is relatively ubiquitous in high-resource settings such as the United Kingdom and they have a high capacity to collect active and passive data. This raises the interesting opportunity to establish a large bank of mental health data from young people that could be accessed by researchers worldwide, but it is important to clarify how to ensure that this is done in an appropriate manner aligned with the values of young people.

**Objective:**

In this study, we discussed the preferences of young people in the United Kingdom regarding the governance, sharing, and use of their mental health data with the establishment of a global data bank in mind. We aimed to determine whether young people want and feel safe to share their mental health data; if so, with whom; and their preferences in doing so.

**Methods:**

Young people (N=46) were provided with 2 modules of educational material about data governance models and background in scientific research. We then conducted 2-hour web-based group sessions using a deliberative democracy methodology to reach a consensus where possible. Findings were analyzed using the framework method.

**Results:**

Young people were generally enthusiastic about contributing data to mental health research. They believed that broader availability of mental health data could be used to discover what improves or worsens mental health and develop new services to support young people. However, this enthusiasm came with many concerns and caveats, including distributed control of access to ensure appropriate use, distributed power, and data management that included diverse representation and sufficient ethical training for applicants and data managers.

**Conclusions:**

Although it is feasible to use smartphones to collect mental health data from young people in the United Kingdom, it is essential to carefully consider the parameters of such a data bank. Addressing and embedding young people’s preferences, including the need for robust procedures regarding how their data are managed, stored, and accessed, will set a solid foundation for establishing any global data bank.

## Introduction

### Young People’s Mental Health in the United Kingdom

Worldwide, 1 in 7 individuals aged 10 to 19 years experiences a mental health disorder, which accounts for 13% of the global burden of disease in this age group according to the World Health Organization [1]. In the United Kingdom, the prevalence of probable mental health disorders in children and young people aged 6 to 16 years has increased from 11.6% in 2017 to 17.4% in 2021 [2]. The most common mental illnesses experienced by children and young people are emotional disorders, namely, anxiety and depressive disorders [3]. Emotional disorders are more common in girls than in boys, potentially getting worse with increasing age to the extent that, in a 2018 UK population-based survey, 22% of young women aged 17 to 19 years met the *ICD-10* (*International Statistical Classification of Diseases, Tenth Revision*) criteria for any emotional disorder, including anxiety disorders, depression, and bipolar disorder [3].

Mental health conditions, including anxiety and depression, often continue into adulthood [4], whereas other potential adverse outcomes of poor mental health in childhood and adolescence include lower educational achievement, self-harm, substance abuse, and violence [5-8].

### Use of Mobile Technology to Capture Mental Health Data

There has been a growing use of web-based resources and mobile technologies to better understand and support mental health. For example, data related to sleep; body movement; or exercise, social connections, and positive activities can be collected via smartphones and wearable devices (smartwatches and sensors). Web-based mental health programs can be used to help remotely monitor patients, send reminders to patients to engage in health-promoting behaviors, or complete electronic standard mental health scales [9,10].

Mobile devices are portable and inexpensive compared to traditional desktop computers and can contain features that provide real-time feedback, although they can drain batteries and use up data quotas [9,10]. Furthermore, new ethical and regulatory issues arise as these technologies develop and evolve, and it is essential to understand the implications of these developments. Because of this, it is more important than ever to research young people’s views on data governance and data sharing.

Previous research has shown that adult study participants are willing to share their data to be used for research by people working to improve health [11-15]. The societal benefit of sharing data arguably outweighs some of the privacy concerns or potential negative consequences, which are described in the following paragraphs [13,15].

Participants’ overall trust in an individual or institution can determine their willingness to share their health data [16]. There have been reservations about government access to data due to issues in the past with governments misusing public data and harming people in vulnerable and minority communities [17]. While one study found participants were accepting of governments accessing their data, it was concluded that this came from a position of resignation—access was considered acceptable because “the government already have all our information” [18].

Previous research has identified discomfort about the sharing of health information with commercial entities [14,15]. A systematic review found that participants were happy to share their data for research as long as the research conducted was not used to discriminate against a specific group of people and was used to help build knowledge, identify issues, and find answers to questions that participants endorsed [18]. Participants suggested that this risk could be mitigated by controlling who has access to health data via a screening process [11,17]. Not all studies have found opposition to government or commercial data access. One study on diabetes research data found that most participants (56%) were happy to allow commercial companies access to their medical history, genetic information, blood test results, and personal information, although it was unclear why this was the case because these findings were not supported with qualitative data [17].

### Rationale and Aims of This Study

Several studies have examined adults’ attitudes toward sharing their health data or genetic data, covering issues surrounding data storage, access to the data, data use, and anonymity of the data. Adults’ views on sharing their data and data governance have been quite consistent across the studies, but we lack evidence of what young people think.

A global mental health data bank containing information about how various factors affect young people’s mental health would provide valuable resources for mental health researchers. Terms of governance acceptable to young people must be established before such a resource is developed.

This study was embedded within the MindKind Study, which was a large, multinational study investigating young people’s preferences for the collection, storage, and sharing of mental health data [19-22]. In the quantitative part of this study, participating young people downloaded and used a mental health app (henceforth, the *MindKind app*), which was used to inform us of young people’s preferences in developing a data bank of information pertaining to young people’s mental health.

The qualitative study described in this paper involved a deliberative democracy approach with young people who had either used or not used the MindKind app to gain a more detailed insight into their preferences for the use of their mental health data. We aimed to determine the preferences of UK youth for a future global data bank of mental health data and capture their views on the collection, storage, and sharing of mental health data.

## Methods

### Ethical Considerations

Ethics approval was granted by the University of Cambridge Department of Psychology Ethics Committee (reference: PRE.2021.031) and the University of Oxford (reference: R73366/RE00). Written informed consent was obtained from each participant before the deliberative democracy sessions, during which the research objectives were described. All transcripts were deidentified, and each participant was given a unique study ID to ensure that they could not be identified. Participants were given a £30 (US $37.63) shopping voucher to compensate them for their time.

### Young People’s Advisory Group

A major underlying principle of the MindKind Study was that young people’s voices should shape the study at all stages [19]. A professional youth advisor was embedded in the study team, worked full time on the project, and ran the young people’s advisory groups (YPAGs). The YPAG was a panel of 15 to 20 young people aged 16 to 24 years with lived experience of mental health difficulties and an interest in mental health who attended fortnightly 2-hour web-based groups to discuss a wide variety of project-related topics. Some discussions were designed to enhance the knowledge or skills of the young people, enabling them to understand and participate in aspects of the research, and others were designed to inform or provide feedback on aspects of the study (including recruitment, educational materials, app design, deliberative democracy sessions, and conceptualization of project outputs).

### Design

This study involved web-based deliberative democracy sessions with young people in the United Kingdom. Sessions were held remotely due to the ongoing COVID-19 pandemic, but this had the additional benefit of more easily being able to reach young people across the United Kingdom.

Deliberative democracy is a qualitative method originally developed in the political field so political decisions could be justified through public deliberation by those who are affected by them [23,24]. It is now used to facilitate discussions outside the realm of politics [25], including engaging communities in complex ethical decisions surrounding emerging technology. It requires that participants be informed, try to understand the perspectives of others, and be willing to work with others who might have different perspectives to reach a consensus [24]. It is distinct from focus groups in that participants are provided with accurate, reliable sources of information to inform the deliberation and involves iterative revision of opinions by participants as they combine information from educational sources with that obtained from the views of other participants [25]. Deliberative democracy was adopted for this study because participants required educational materials before the session so that they would be on a level playing field in the sessions.

The sessions generated *deliberative outputs* consisting of the group consensus on the issue discussed, which should be recognizable to and ratified by the participants involved in the deliberative democracy session, and *analytic outputs* generated through subsequent analysis of event data based on scientific inquiry [24]. In this study, the deliberative outputs would comprise the group consensus regarding participants’ preferences for a data governance model, whereas the analytic outputs would result from the qualitative analysis of why they had those preferences.

### Sampling and Recruitment

Participants in the study were young people aged 16 to 24 years at the time of enrollment recruited from across the United Kingdom. Participants had lived experience of anxiety or depression as ascertained by a positive response to any of the following eligibility questions:

1. Have you ever felt that you could have benefited or did benefit from access to support for anxiety or depression?
2. Have you witnessed or experienced anxiety or depression within your family or close friends?
3. Do you have a strong interest in anxiety or depression?

We advertised the study via paid social media advertisements (eg, on Facebook and Instagram). We also used our own networks, including departmental Twitter (subsequently rebranded X) pages and Instagram accounts, and placed posters in our communities in Cambridge, Oxford, and Kent. Individuals who wished to participate or learn more about the study contacted the UK study team via email. We recruited 2 groups of participants. *App-naïve participants* were those who had not downloaded and used the MindKind app and were recruited primarily via social media. *Coenrolled participants* were those who had previously downloaded and interacted with the MindKind app, and they received a pop-up notification after 2 weeks in the study asking whether they would like to sign up to join deliberative democracy sessions.

A member of the research team was in regular contact with participants to answer any questions that they had about the study and to arrange the times for the web-based sessions. All participants were provided with a participant information sheet and completed a web-based consent form and a sociodemographic survey to confirm study eligibility and assess the diversity of the sample. Each session’s participants were unique and attended only 1 deliberative democracy session.

### Procedure

#### Educational Material

The MindKind Study developed 2 animated videos, which were coproduced with YPAGs based in the United Kingdom, India, and South Africa and voiced by our professional youth advisor [21,22]. The educational videos were sent to participants 1 to 2 weeks before their scheduled session to ensure that participants had a basic understanding of the topics to be covered.

The first video was approximately 10 minutes long and provided a brief overview of the proposed global mental health data bank and a brief introduction to each of the 7 questions that participants would be asked to deliberate on. The second video was approximately 25 minutes long and described 4 possible data governance models that could apply to a future data bank.

#### The Deliberative Democracy Process

A topic guide (Multimedia Appendix 1) and a set of facilitation slides were developed in collaboration with the YPAGs to address the 7 key questions as part of the MindKind Study. The questions (Multimedia Appendix 2) were (1) who can access the data? (2) Where are the data hosted? (3) Who controls the data? (4) What do people have to do before they can access the data? (5) Who takes on the cost of managing the data? (6) How can people see the data? (7) What kind of research can people do with the data? [22].

Deliberative democracy sessions were stratified by previous exposure to the MindKind app (ie, naïve or coenrolled participants). Where possible, we arranged sessions with different age groups to ensure that younger participants would feel comfortable. The process is further described in the MindKind Study protocol [22] and a paper describing the MindKind adaptations for conducting public deliberation using digital platforms [20].

Each web-based deliberative democracy session was facilitated by 2 trained members of the study team and lasted approximately 2 hours including 2 short breaks. Sessions were held on the Zoom videoconferencing platform (Zoom Video Communications) using Otter AI (Otter.ai, Inc) to provide automated closed captions. Participants were able to contribute to the discussion by speaking and using the chat feature to make comments or ask the cofacilitator any questions. To ensure a smooth discussion and that all voices could be heard, the cofacilitator verbalized comments from the chat box.

At the start of each session, participants were provided with the terms of engagement, which encouraged active and respectful participation. Participants were reminded that their privacy would be protected, they did not have to answer questions if they did not want to, and they could withdraw from the study at any time.

The session facilitator presented the facilitation slides described previously, which addressed the 7 questions in turn. Attendees had previously learned about these questions via the presession educational material. The group discussion addressed each of the 7 questions, with participants giving their views as to whether each option was seen as acceptable, unacceptable, or somewhere in between. Participants were free to speak or raise their hands to contribute, and the facilitator prompted participants who had not spoken on each question.

Immediately, during and after the session, a member of the research team collated the deliberative outputs from the session, creating a table classifying governance options as acceptable, maybe acceptable, or unacceptable. Session audio recordings were transcribed and anonymized, and the written content from the chat box was integrated into the appropriate parts of the transcript.

### Analysis

We applied the framework method of thematic analysis [26,27]. Initially, 5 transcripts were allocated to different members of the research team (AMB, BF, EC, FA, and LN). Team members independently read the transcripts; familiarized themselves with the data; and highlighted salient points, which were open coded by each researcher. These excerpts were put into a Excel (Microsoft Corp) spreadsheet. Team members then met to compare the labels applied to their own transcripts and agree on a set of codes to be used for subsequent coding. We added a short summary description to each of the codes generated, forming a working analytic framework.

The transcripts and working framework were entered into NVivo (version 12; QSR International), and this file was placed on a shared drive. In total, 5 members of the team (AMB, BF, EC, FA, and LN) were allocated 2 transcripts each, which they coded line by line to the thematic framework. Researchers created additional codes where they were required during a series of meetings to discuss the coding process.

Once all the transcripts were coded, we generated a matrix framework that was then exported to Microsoft Excel. We charted the data by summarizing verbatim text and making analytical notes. The framework matrix enabled us to identify characteristics of the data and any group differences according to age or app exposure. Regular team meetings were held to ensure charting reliability and interpret the patterns between the themes and subthemes.

## Results

### Sample

A total of 11 web-based deliberative democracy sessions were held between August 2021 and April 2022 with 46 young people aged 16 to 24 years (median age 19 years). Of the 46 participants, 22 (48%) were *naïve participants* who had not used the app, and 24 (52%) were *coenrolled participants* who had previously used the app (see Table 1 for more details). Due to significant difficulties in recruitment, including many who did not attend agreed sessions, some group sizes were smaller than anticipated.

**Table 1:** Participants who attended each deliberative democracy session.

Session number	Participants, n (%)	Age of participants (years)	App exposure
1	3 (7)	16-17	No
2	4 (9)	16-18	No
3	3 (7)	19-23	No
4	7 (15)	19-23	No
5	6 (13)	16-18	No
6	4 (9)	16-18	Yes
7	2 (4)	16	Yes
8	2 (4)	18-19	Yes
9	4 (9)	19-22	Yes
10	4 (9)	19-22	Yes
11	7 (15)	18-23	Yes

The sociodemographic characteristics of the coenrolled and naïve samples are shown in Table 2.

**Table 2:** Sociodemographic characteristics of the sample^a^.

		Non–app users	App users
**Gender, n (%)**			
	Woman^b^	18 (82)	18 (75)
**Ethnic background, n (%)**			
	Non-White^c^	11 (50)	10 (42)
	White	10 (45)	13 (54)
**Current stage in life**			
	Secondary school or college, n (%)	10 (45)	8 (33)
	University, n (%)	7 (32)	10 (42)
	Not in education^d^	<5	<5
**Illness or disability, n (%)^e^**			
	Yes	5 (23)	11 (46)
	No	16 (73)	12 (50)

^a^One participant chose prefer not to answer and is not included in the table.

^b^Others were male or nonbinary.

^c^Non-White participants were of South Asian, East Asian, Black, and mixed or multiple ethnicity.

^d^This group includes those working or not in employment or education combined to avoid accidental disclosure.

^e^Where values do not add up, the participants declined to answer.

### Themes

**Figure 1:**
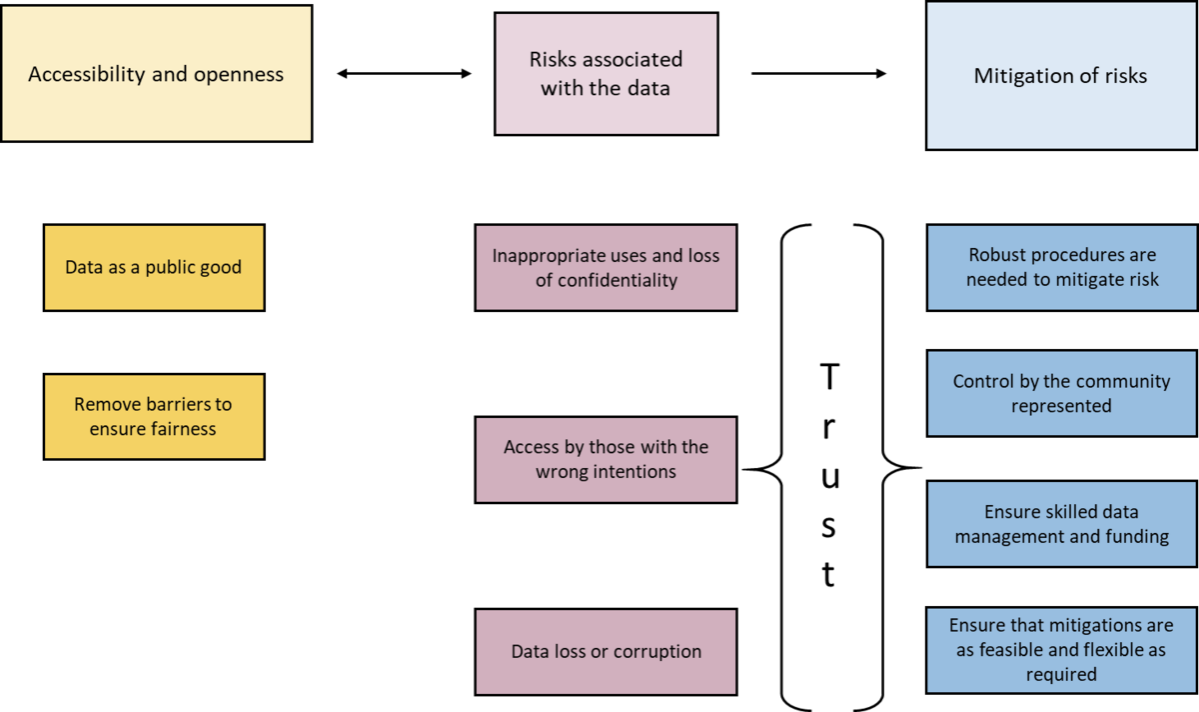
Themes and subthemes generated from the analysis.

Three overarching and interrelated themes were identified by participants as key to increasing trust and participation in the establishment of a global mental health data bank: (1) *accessibility and openness*, (2) *risks associated with the data*, and (3) *mitigation of risks*. The themes and subthemes are summarized in Figure 1 and detailed in the following sections; quotes are provided to illustrate the themes.

#### Theme 1: Accessibility and Openness

##### Data as a Public Good

Young people believed that establishing a mental health data bank was acceptable. Typically, young people expressed hope that such a resource could be used to empower those with mental health difficulties and contribute to research to improve their lives. Typically, they wanted their data to be as accessible as possible while mitigating potential risks for data misuse and protecting confidentiality. This was driven in part by the belief that sharing data more widely would allow for more research, creativity, and discovery. Furthermore, young people believed that data could be used to empower communities by improving the understanding of mental health difficulties:

My idea for who can access the data, if it was mental health data, I thought it would be good for teenagers to be able to access it, because then they’ll be able to understand mental health.

FG2; aged 16-18 years

##### Removing Barriers to Ensure Fairness

Participants talked extensively about removing barriers that could prevent certain groups from being able to access the data. One of the primary perceived barriers to fair data access was cost—both direct and indirect. Young people understood that the costs of storing and managing data would need to be covered, but most were concerned that, if this cost were to be met by those accessing the data, individual researchers with less access to funding might be unable to conduct the research they want:

I don’t know if classist is the right word to say, but like it makes it so that people with money are able to access the data and people that don’t have as much money can’t access the data.

FG4; aged 19-22 years

Some young people preferred that costs be met by those who access the data but with flexible charges to avoid barriers to access. For example, those with less funding could pay less than those with more funding using a sliding scale. Others thought that cost could depend on how much data people needed, how long people wanted to use the data for, or the complexity of the analysis proposed.

In general, charging at a macro level was preferred (ie, an organization, institution, or government) because it was thought not to preclude researchers without the funds from data access.

Participants were also concerned about indirect costs—for example, some thought that requiring ID for data access might result in unacceptable costs for individuals in countries where IDs can only be obtained by paying and wanted to ensure that the data could be accessible wherever a researcher might reside. Young people also wanted to avoid nonfinancial barriers to data access, such as limiting access to data to researchers in certain countries:

I agree, don’t want to gatekeep by ability to pay for ID (as someone who couldn’t afford a passport for many years!).

FG4; aged 19-22 years

I feel like nowhere in the world should the data not be reached just because you’re from a certain country. It’s kind of unfair.

FG5; aged 16-18 years

#### Theme 2: Risks Associated With the Data

##### Inappropriate Uses and Loss of Confidentiality

While young people supported the equitable, open access of data, they had strong opinions on mitigating the risk of misuse and whom they would trust with their data. For example, many expressed concerns about how countries with strong societal stigma against those with mental health problems or with a poor track record on human rights issues might manage and use the data. Concerns were raised about the risk of identification and the harm that this could cause to individuals:

I think the only place I wouldn’t want my data going is North Korea...because they don’t have the best reputation for being not-harmful.

FG6; aged 16-18 years

##### Access by Those With the Wrong Intentions

The intentions of those accessing the data were important to young people. Private companies were viewed with some skepticism and described by young people as self-serving and profit-oriented and might misuse data to these ends:

There have been instances in research where the way that—even the way that data is interpreted will be biased to give the outcome that the people involve want, especially in for-profit studies. So I guess that would be a concern for me.

FG1; aged 16-17 years

Some voiced concerns about a poor track record for security among private companies and specifically distrusted advertising companies, insurance companies, and “big pharma” to protect their data. The media were also perceived as untrustworthy and likely to exaggerate or misinterpret findings for profit.

While researchers were generally seen as having better intentions for the data, some participants highlighted that researchers can also have bad intentions and their research can have harmful consequences. Specifically, they wished to limit any research that actively harms or has the potential to harm vulnerable and marginalized groups:

One thing I had in mind when I saw this question is there’s a book called “The Bell Curve”...but some researcher took a bunch of data about—I think it was IQ scores, and it was supposed to be a big mental health thing, but it just ended up being a bunch of racist crap. So, he was a researcher and he had proper access to the data...he will have passed the first six questions about all the other controls, but I think if you put question seven to him about what type of analysis, what are you using the data for, I think he would have failed that. So that would probably be most important for me.

FG8; aged 18-19 years

One participant highlighted the potential for genetic data contributing to research that might then be applied in eugenics, giving rise to some young people suggesting that research with potentially damaging consequences should receive a higher level of scrutiny.

##### Data Loss or Corruption

Beyond these concerns about how the data could be used to cause harm, young people were also concerned about the sabotage, corruption, and destruction of their data. For example, if the data were downloadable, they could be altered and false information could be shared, whereas malicious individuals could sell or leak the data or deliberately destroy them entirely. Young people suggested that having multiple people controlling the data and storing the data in multiple locations could reduce the risk of intentional loss or corruption despite the potential for loss of complete control over the data:

My only concern is how safe the data is, how it’s encrypt[ed]. Because some people could just hack into a server and just corrupt the whole data.

FG9; aged 19-22 years

#### Theme 3: Mitigation of Risks

##### Robust Procedures Are Needed to Mitigate Risk

Participants were keen to ensure that risks involving loss, corruption, or misuse of data were mitigated in various ways. Ethical use of data was seen as fundamentally important, and young people emphasized the need for robust ethics procedures. Ensuring that data are used ethically was seen as one of the most important functions of a review board. Some ethical training was seen by most as crucial. Some young people wanted mandatory ethics training for all applicants, with exemptions issued to those who already have a good knowledge of ethical data use. Young people wanted this training to be robust and not a “tick-box” exercise, with many participants proposing a thorough ethics assessment after training:

There should be an assessment at the end of it that’s like a proper assessment, not just like an online quiz, multiple choice...Like something maybe scenario based or something where they actually have to put themselves in the position of something that could go wrong in the databank and what they’d do or what they’re allowed to do and what they can’t do, and it’s actually not just like closed responses.

FG4; aged 19-22 years

As well as being concerned about the data being used ethically, young people wanted to ensure that the data themselves were reliable. The concept of recreated data was raised by researchers as a method to protect confidentiality. A recreated data set is a data set that reflects key aspects of the original data but has been modified and combined in such a way that data points no longer reflect specific individuals. Many young people expressed concern that use of a recreated data set might iron out nuances and granularity in the data, which could have serious real-life implications by distorting research findings.

Maintaining privacy, anonymity, and confidentiality was seen as fundamentally important. Thorough anonymization enabled young people to feel safer—even if data were to be misused as this would not have an individual, targeted impact. Data that could lead to identification could be used to discriminate or stigmatize a specific person. In addition, answers to other questions about data access were more liberal on the understanding that the data would be thoroughly deidentified. Some young people remained skeptical that true anonymization was possible, particularly for individuals from small or niche communities. This occasionally led young people to prefer a recreated data set, but the aforementioned concerns about reliability tended to outweigh this:

If someone has two intersectionalities like they’re black and gay and the dataset is recreated and it only takes into account one of them because it averages it out, it sort of ignores the really small...pockets of people in that dataset.

FG4; aged 19-23 years

In light of these concerns, some participants proposed flexibility in level of access depending on individual needs, expertise, intentions, and requirements. Under such a system, only properly trained researchers with clearly positive intentions who had a true need to access the most sensitive, granular data would be granted access to it. Other researchers may have access to less sensitive, aggregated, or recreated data without such stringent requirements. Some young people also proposed that the most sensitive, confidential data should be stored in only one, well-protected location, with less sensitive or confidential data stored in other places.

##### Control by the Community Represented

Participants reported that it is important to retain some control over the data. Most young people did not want the data to be available to just anyone but instead believed that some kind of vetting process was important, such as assessing a person’s intentions for using the data and that they could be trusted to do this, providing them with the required training, and potentially asking them to sign a legal contract. Young people wanted to ensure that a group of people were responsible for this process of determining who could access their data. Having the entire community vote was seen as ideologically desirable but practically infeasible, so young people tended to see a community review panel who would make decisions about who should be able to access their data as a good option:

I think that the only way that you can really represent a community is that if you let that community speak for itself, and that is actually part of the reason why I don’t like community hires a manager either, because I don’t really think it’s feasible a whole group of people to be represented by one person.

FG7; aged 16 years

Given their desire for vetting, young people tended to view data download as unacceptable because once data were downloaded, they could be freely shared with people who have not gone through this procedure. Some young people proposed a more flexible approach whereby a recreated data set, aggregated data, or a subset of less sensitive data was available for download but a more thorough procedure would be necessary to access the true, granular data. Young people generally approved of data access via a server and proposed a high level of security for the server to avoid hacking:

You’d have to make sure that that server was extremely well protected and able to fend some attacks from people who may wish to hack into it and use that data in a negative way.

FG9; aged 19-22 years

A single data manager was viewed as unacceptably risky, and young people were keen to distribute power among a group of people with a personal stake in the data. Distributed power over the data bank would avoid any one person’s biases becoming entrenched in the system. Some young people also wanted multiple organizations or governments funding the data as they were concerned that a sole funder could seize control or misuse the data. A few participants also mentioned that storing data in multiple locations would aid the distribution of power:

If it’s just like one person or like one or two people, that’s only one set of biases and their biases sort of control the whole thing, if that makes sense. Whereas if it’s a community or like tens of people, then their biases counteract each other.

FG4; aged 19-23 years

Young people generally supported procedures to hold anyone who misused the data accountable, such as using a formal contract. Funders and managers of the data should also be held to account to the same standards as anyone who accesses it:

I think that [signing a contract is] important because it will deter people from doing anything they shouldn’t be doing with the data, and then even if they do something bad, you can sue them. So, it’s not perfect—you can still do bad things—but at least they’re held accountable for it.

FG3; aged 19-23 years

##### Ensure Skilled Data Management and Funding

Another way to mitigate risks of data misuse was to stipulate that the data be accessed by the “right” people with appropriate skill levels. Participants were concerned that sensitive data should only be accessible to suitably trained professional researchers. However, even sharing completely deidentified data was considered to be risky as harm beyond identification is possible. For example, young people were concerned that the press (in particular tabloid media) could distort findings in a way that is harmful to youth mental health generally. Some young people proposed flexible systems with different levels of access depending on individual expertise and intentions:

I guess people working in a newspaper would have an interest to try and make it controversial or like make a finding out of just generic data.

FG4; aged 19-23 years

If a normal person that’s not part of the mental health medical field, they should just see maybe an infographic or maybe just some paragraphs that conclude what’s in the data so they’re aware, globally, of mental health.

FG1; aged 16-17 years

Researchers at universities and other non–profit-motivated organizations were perceived to have an interest in conducting research properly and ensuring that the findings were accurate, as opposed to private companies, who were seen as primarily motivated by profit. Some—but not all—young people placed the government in a similar category with an interest in the public good. Others presented a more nuanced view:

Whether a government should fund the databank depends on whether the government is democratic or not, or even if they are democratic, whether they’re corrupt or not.

FG7; aged 16 years

Most participants trusted charities and nongovernmental organizations, although others listed specific named charities that they considered not to be trustworthy because of a track record of stigmatization or harm:

I wouldn’t want somebody like [named charity] holding my data, because I don’t like them.

FG6; aged 16-18 years

Mental health care practitioners were seen as motivated to improve the mental health of young people and having the appropriate skills to use the data well. Young people wanted those with skills in data dissemination to have access so that findings could be relayed back to relevant communities:

Data-analysis-wise, maybe you need some data scientists which may not be very good at psychological stuff. So maybe psychiatrists maybe need to work with data scientists to interpret data.

FG9; aged 19-22 years

The intentions of those accessing the data were seen as important while acknowledging that even well-intentioned use could cause harm. Young people wanted those who accessed the data to be working for the benefit of society and in particular to improve the health of young people. Profit was not an appropriate intention for most participants, although it was acknowledged that profit-making is not inherently harmful. Particular concern arose about intentions that could harm, for example, developing targeted advertising to encourage harmful coping mechanisms, stigmatizing mental health problems, or charging individuals higher life insurance premiums:

I was also going to say, yeah, getting targeted ads based on the stuff that you look at, the last thing you really want is them being able to have access to private data about things like, for example, your mental health, because the last thing you want is something saying, “Oh, are you struggling from depression? Try Jack Daniel’s whiskey.”

FG11; aged 18-23 years

Skilled data management was also seen as very important; young people proposed that the data managers should have expertise in confidentiality, with more trust that privacy would be maintained if data were managed by a group of skilled, reliable individuals. Some suggested that the review panel should represent a variety of skills (eg, psychologists, students, and activists), and others thought that different decisions should be made by people with specific expertise in that area:

In the review panel there could be a few psychologists, some students as in college/university students, researchers who don’t necessarily have a degree or any kind of certificate in psychology, but they are known for research. And then...you know activists, like mental health activists.

FG5; aged 16-18 years

Young people were also keen that the data bank be funded by individuals with the correct intentions. They often questioned why an organization or government would volunteer to spend money on a data bank. Some young people felt that the government has a duty to improve mental health and were therefore obvious funders for a data bank. Universities were generally viewed as trusted institutions with an interest in furthering knowledge as well as having research expertise and thorough ethics review processes:

If the research can help the general public, then the government should be contributing towards that, just like they give money for the NHS.

FG3; aged 19-23 years

##### Ensure That Mitigations Are as Feasible and Flexible as Required

Young people acknowledged that accounting for feasibility, practicality, and financial viability was necessary. One frequent concern was the global nature of the proposed data bank. This means that the data bank would include data collected from individuals in countries with varying data protection laws. Some young people considered that the lack of certain laws applying to their data would make them feel unsafe. Others were more concerned about how these differences could be resolved practically. Some participants argued that, if data are held and managed in the United Kingdom, other countries should abide by the UK laws or rules but with agreement that one country having more control than the others could create an unfair power dynamic:

I’d be worried about GDPR [General Data Protection Regulation], especially if one country had it and another country had their GDPR regulations different to the other countries...so they might have different rules of how they store the data.

FG2; aged 16-18 years

A few young people were concerned enough about differences in global policy and attitudes that they did not think that a truly global data bank was feasible, whereas others believed that a global data bank would be a valuable asset to global mental health research and that challenges could be managed.

Other practical concerns regarding a global data bank revolved around the ability to hold people to account for misuse of data. Variations in professional expertise worldwide were also of concern. Language barriers, currency conversion, and differences in ID worldwide were also flagged as issues that would need to be resolved when establishing a global data bank. Young people felt that having data managed by “real people” could allow for flexible systems that mitigate some of these concerns (eg, those without ID could be vetted more thoroughly in a different way):

If anything did happen and, for some reason, that contract was broken, where would it go to? Would you have a trial in your own country or would it have to go to an international court or the European courts, or somewhere like that?

FG9; aged 19-22 years

Practicalities were often in tension with young people’s ideological preferences. For example, young people favored the democracy afforded by consultation on data management with the entire community as the most representative option. However, practical concerns here were numerous—it would take a great deal of effort and may become bureaucratic, a vocal minority might be heard over a quieter majority, and the community may become disengaged. For these practical reasons, participants proposed a community review panel as a more viable option that distributed power among a group. Some young people proposed that a community review panel should be combined with the community deciding—for example, the panel could be elected by the community, or the community could be given a yes or no vote with nuances decided by the panel. Some participants suggested that the most controversial decisions should be made by the entire community, whereas less potentially harmful uses of data could be approved by a panel. Others argued that less controversial studies could be approved by an algorithm. Young people stipulated that a review panel should be as representative as possible of the entire community to compromise between the practical benefits of a review panel and the ideological desire to represent the entire community well:

I think the community decides is pretty unacceptable is that because in a community, the most vocal people tend to have the largest say and it can end up becoming a vocal minority who have really strong opinions on certain things.

FG10; aged 19-22 years

Similarly, young people were against the idea of charging for access to data. However, they acknowledged that it might be the most feasible option to cover the operational costs of the data bank. Nevertheless, they believed that institutions, rather than individuals, should primarily bear the cost:

I’m all for having people pay money...I know that there are some health databases that people pay money to access for research purposes, and I think that would help cut down costs.

FG10; aged 19-22 years

Similarly, young people thought that a secure server would provide the best protection for their data but equally saw that this might limit the ability of researchers to conduct their preferred analysis or be unreliable in parts of the world with inconsistent power or internet connection:

I do believe, for practical reasons, it is better to download the data from the researcher’s point of view. Just from personal experience, doing things in the server can be very tedious and very chaotic.

FG8; aged 18-19 years

Despite strong support for robust ethics training, some young people were concerned that the practical hurdle might lead people to seek out lower-quality, less scrupulous data sets. Some proposed flexible systems whereby only research with a high risk of harm or controversy would need thorough ethics training and assessment.

Ensuring that a data bank could continue in the long term was important to participants, with contrasting views on how the data bank should be managed and by whom. While it could be more practical for one organization or group to manage the data, multiple funders could ensure sustainability. Others suggested that having governments fund the data bank could also provide this consistency or resource. Participants were concerned about what would happen to the data in the long term, where data access becomes less stringently monitored as the data age (and lose some of the sensitivity and identifiability of recently collected data). It was seen as practically unviable to maintain a review panel in the longer term:

I was thinking that a review panel might not end up being useful in the long run, because I was thinking, over time, it would be harder to find enough people who are actually willing to review it. So maybe a manager might be easier after a while.

FG10; aged 19-22 years

## Discussion

### Principal Findings

We ran 11 deliberative democracy discussions of 7 questions related to the governance of a potential global mental health data bank among young people aged 16 to 22 years in the United Kingdom as the vast majority of research to date has focused on adults. Participants were enthusiastic about data being as accessible as possible to a variety of people because data access was seen as a public good. Our broader, global study found some differences by region, so we report findings specific to the United Kingdom [21].

Young people were also very enthusiastic about access determinants being equitable, but this desire for accessible data was in tension with concerns about risks associated with data use. In particular, young people wanted to limit inappropriate or badly intentioned use of their data, ensure that their privacy was maintained, and prevent the sharing of unreliable data. There was enthusiasm for various risk mitigation strategies to counter these possibilities. Young people wanted robust procedures to ensure ethical use of data and the ability to hold people to account for misuse. Control of the data by the community who contributed to them was important, as were distributed power and diversity of those in charge of the data. Young people wanted to ensure that these mitigations were both practical and flexible; mitigations were a necessary means to ensure that data would be accessed by those who could be trusted to use them skillfully, with the right intentions, and with positive consequences.

### Implications for Policy

Our finding that young people were generally positive about the idea of widely sharing mental health data was in keeping with those of previous research suggesting that adults are willing to share their data for research purposes [11-15] and perceived that the societal benefits of this outweighed potential negative consequences [13,15]. While previous research has also suggested that participants want their data to be as useful as possible via increased access, the young people in this study framed fair and equal access to data as a good in itself rather than suggesting that fair access is simply a means to maximize the public good of data availability.

The desire to obtain equitable access and maximize public good led to many interesting discussions about how to remove barriers such as cost. Cost was especially interesting as it affected young people’s approach to all aspects of the discussion—for example, requiring researchers to provide ID was perceived as ideal in terms of controlling data and limiting access by those with poor intentions but might unfairly affect researchers from countries or institutions with less money or those who are not affiliated with an institution. This has broad implications for policy as it highlights the need to consider both direct and indirect costs as a cause of potential injustice in data access.

The intention behind data access application was an important consideration consistent with the findings of other studies [12]. Participants were especially concerned about the uses of data that could increase stigma or prejudice against certain groups of people, echoing the findings of Breakey and Dipinto [18]. There is an obvious tension here with the desire for research to be shared fairly and freely, especially when young people mentioned specific countries as being untrustworthy. Young people recognized that the presence of an untrustworthy government does not imply that the people of a country are themselves untrustworthy, but in practice, it would be challenging to allow free access to citizens and prevent misuse of data by their government.

Previous studies have shown that people are willing to share their physical health data with commercial entities [17]. In contrast, our participants were cautious about sharing their data with commercial organizations and suspicious of profit-driven interest in using their data. This may be because the participants in our study were younger or because data about physical health conditions are seen as less sensitive than mental health data. Our participants expressed strong views on sharing their data with commercial organizations even though many young people might regularly, and possibly inadvertently, share personal data with for-profit social media companies and health apps. Future research could explore directly this disconnect between principles and practice.

In addition to limiting who could access data by their identity or profession, young people were keen on robust ethical procedures. The level of modular ethical training and assessment proposed by young people in this study was interesting insofar as it was significantly beyond what is typically required by researchers applying to access data. However, the reasoning of young people in this study was sound—researchers from different institutions and worldwide locations may have very different previous training in ethics, and conducting additional training could standardize abilities. Ajuwon and Kass [28] conducted a research ethics training workshop with researchers and reported significantly improved understanding of research ethics in a follow-up test, which indicates that, even among trained researchers, additional training can improve and standardize skills.

Young people understood that data would not be attached to such identifiers as their name or national insurance number but remained concerned that, when a large number of variables exist for an individual, reidentification may be possible. This concern has been explored in relation to research on rare diseases, where true deidentification may not be compatible with accurate research findings [29]. Participants in this study proposed tiered access systems as a way to reduce identifiability, for example, access to full data only for those who need such access and have undergone very stringent training, with easier access to aggregated or synthetic data. While this adds administrative complications to the sharing of data, it would allow for wider access while allaying concerns about privacy. As others have observed in adults, young people expressed a significant preference for data access to be via a secure server; most young people were concerned that downloading would decrease the security of their data, resulting in a loss of control and potential misuse [11,17].

Even with mitigations in place about who can access the data and within the data themselves, participants wanted the participating communities to retain control over their data. While complete democracy was often seen as infeasible, a review panel of either volunteers or paid community members was viewed as a good compromise, which echoes the findings of other research [12]. However, this finding conflicts with results from the quantitative arm of the MindKind Study, which found that UK participants preferred democracy or a professional review panel over a voluntary panel [21]. This may be because of the extensive educational materials provided to those in the qualitative arm of the study or because of more subtle differences in the phrasing of options.

In addition, our participants stressed the importance of diverse representation of their community and distribution of control and funding, which supports findings from previous research that, as well as being representatives from the community, participants want members of a panel controlling data to have skills in research [12,17]. Our research demonstrated that young people in the United Kingdom believe that distributing power among a range of individuals with different skills is the best way to ensure the competent and representative management of their data. Finally, young people tempered their ideological views with practicality.

### Strengths and Limitations

This study uniquely provided insights into the views of young people in the United Kingdom on the governance of mental health data. We acknowledge that this qualitative study used a convenience sample and, therefore, may not represent the views of other young people.

The main limitation of this study was that some of the sessions had a small number of participants; our group sizes ranged from 2 to 7 participants as we considered it important to proceed with planned sessions despite last-minute cancellations. Smaller groups will have limited the range of experience and opinions expressed. Nevertheless, we found that the smaller groups were cohesive and the participants engaged well and contributed their ideas freely. Participants had the time to speak more in depth about their ideas and justify their reasoning, were able to deliberate the questions, and respected each other’s opinions and ideas. In addition, across the entire study, there was a diverse group of participants in terms of ethnicity and age, and therefore, we were able to capture a wide range of life experiences across the sessions. We found that, regardless of session size, participants tended to say similar things between sessions and that the consensus reached did not seem to vary greatly. While this study is a relatively preliminary exploration of data governance preferences among young people, we were struck by the consistency of themes and consensus building, which did suggest that the data captured accurately reflect the opinions of young people in the United Kingdom.

Deliberative democracy sessions are usually conducted face-to-face; however, we conducted our sessions digitally using videoconferencing [20]. We encountered technical challenges such as poor broadband connection or microphone issues that limited some participants’ contributions to the discussions. Because the sessions were web based, this may have made it easier for participants to not attend at the last minute. Grönlund et al [30] compared web-based deliberative democracy with face-to-face deliberative democracy and found that participants were able to engage in high-quality deliberation when technology performed properly.

One strength of conducting the sessions digitally was that participants had a range of methods they could use to communicate; we made use of the chat and reaction emojis throughout the sessions. In addition, this feature was beneficial for those who felt less comfortable speaking out loud as they were still able to share their ideas. Digital sessions also extended our geographical range. Participants could take part in the sessions regardless of where they lived in the United Kingdom and could choose from a number of time options. This was beneficial as it enhanced the findings and made them potentially more representative of the entire population with the addition of diversity and, therefore, life experience to the groups.

The coproduced educational materials were helpful for participants to grasp some difficult scientific concepts. However, there was one concept (a “recreated dataset”) that proved a difficult term to convey to young people, which meant that it was difficult to have a meaningful discussion about it [31].

### Conclusions

We found that young people in the United Kingdom are able and willing to successfully prepare for and participate in public deliberation about the sharing of their mental health data. While the web-based setting made this more challenging in some ways, it also brought potential benefits for the diversity of our sample and the accessibility of the research. Young people had many concerns about the possibility of misuse and negative consequences of sharing their data but were able to suggest mitigations to these concerns and were broadly positive about the collection, sharing, and use of mental health data by researchers and other relevant, vetted stakeholders to improve mental health and well-being. A global mental health data bank is acceptable to young people in the United Kingdom and would provide significant resources for future research.

## Appendix

Multimedia Appendix 1 Topic guide.

Multimedia Appendix 2 Seven key questions.

## Abbreviations

ICD-10: International Statistical Classification of Diseases, Tenth Revision
YPAG: young people’s advisory group

## Data Availability

The data sets generated and analyzed during this study are available from the corresponding author on reasonable request.
