# Supplementary material for: Preferences on Governance Models for Mental Health Data: Qualitative Study With Young People
Source: JMIR Form Res. 2024 Apr 23;8:e50368. doi: 10.2196/50368 (PMC11077411; doi:10.2196/50368)
Supplement: Multimedia Appendix 1 [file formative_v8i1e50368_app1.docx]

MindKind Community Sessions: Facilitation Guide

1. **Terms of Agreement (Ground Rules the group agrees on)**

- Listen to and respect each other’s perspectives.
- Keep the information shared confidential.
- Discussion is being recorded for transcription purposes, but participants may not record, take photo or video, or take screenshots during the conversation.
- Do not interrupt each other.
- Participants are welcome to leave to attend to their needs.
- If participants do not want to verbally share an idea, they may write it in the chat box [if available] or send a message to the facilitator.
- [Discuss options for “hand raising” or other functionality based on platform.]
- [Discuss with participants: preferences on video on vs. off, muting.]
- [Additional ground rule suggestions from participants.]

1. **Icebreaker:** introductions in a line or two followed by an icebreaker activity.
2. **Recap of educational materials**

*The facilitator will take the participants through a brief recap of educational materials, that is, the four animal models, their characteristics, and how they are described across the seven ‘questions’. Before the recap, facilitator sets expectations for the upcoming poll of their preferred model.*

1. **Things to remember**

*Before the discussion begins, the facilitator will emphasize that there are no right or wrong answers and that the purpose of the discussion is to understand what the group thinks and feels about sharing their mental health data. Also, participants will be reminded that they do not have to restrict themselves to the four models; they can make their own by picking features that they like. The facilitator will reiterate this throughout the discussion as required.*

1. **Favourite Model Poll: pick your most preferred model**

*This is the first question asked to the group. Participants will pick one favourite model; this will be anonymous. An additional ‘none of the above’ option will be available, which does not confine the participants to just the four models.*

*When the poll is closed, the second facilitator will note the model that is most picked and also least picked. The discussion will open with the following probes,*

**Fav Model: What do you like the most about it? What do you not like?**

**Least Fav Model: What do you not like about it? Is there something you like?**

1. **Discussion of each component/feature:**

*Each of the ‘seven questions’ will be discussed. The second facilitator will capture pointers from the discussion on the slide in real time.*

- 1. **Who can access the data**

Anyone, people with certain jobs, people with certain skills, people from certain places

*Who do you think should have access to young people’s mental health data, why?*

*Who do you think should not have access to young people’s mental health data, why?*

**Task as a group: Put the groups of people (i.e. anyone, people with certain jobs) into one of three columns i.e., acceptable/maybe/unacceptable for accessing data.** *Through this task, benefits, harms and concerns can be elicited.*

- 1. **Where is the data hosted**

One place, many places

*Facilitator gives example of how one/many places of storage would translate in real life settings.*

**Where should the data be hosted? Why?**

- 1. **Who controls the data**

No one, community decides, community review panel, community hires manager *(may need to explain what is meant by ‘controls’)*

**What are your concerns about who controls the data?**

**What are the advantages/disadvantages of each choice?**

- 1. **What do people have to do before they can access the data**

Ethics training, provide ID, review board approval, sign contract, pay money

**What should be the process people need to follow to access this data? What can be the steps of this process? Why are these necessary?**

- 1. **Who takes on the cost of managing the data**

People who access it, government, organisation/institution, private company

*What will be the implications of who takes on this cost?*

**What are the advantages/disadvantages of who takes on this cost?**

**Who should take on/not take on this cost, why?**

- 1. **How can people see the data**

Download it, view it in a server, view a recreated dataset

**How should people be able to see this data? What made you choose your answer?**

**What are the advantages/disadvantages of the different mediums?**

- 1. **What kind of research can people do on the data**

Anything, certain types of analysis, certain types of projects

**What kind of research should be allowed with this? Why?**

**What should not be allowed, why?**

**What are your concerns about this?**

1. **Group’s model: preferences from discussion for consensus.**

*The second facilitator/note-taker puts a table on the slide with the preferred set of features (based on the output from the above discussion) for the group to see and comment on. The second facilitator also summarizes, giving the group time to think one last time before the discussion closes. Could use the raise hand feature for consensus.*

*The facilitators will highlight group’s agreement/disagreement on the preferences.*

1. **Group’s non-negotiables for the model** (E.g. “It is unacceptable to us to have a databank hosted in one place.”)

*The facilitator tries to capture what the group thinks is unacceptable when it comes to their use/access/sharing of their mental health data in terms of a global mental health databank.*

*The facilitators will highlight group’s agreement/disagreement on the unacceptable features.*

1. **Closing Polls:**
   1. **What is your most important organizing question from the discussed 7 questions?**
   2. **What is your least important organizing question from the discussed 7 questions?**

*The participants will answer these two polls before the discussion closes.*

1. **Share link to exit survey**
