# Supplementary material for: Preferences on Governance Models for Mental Health Data: Qualitative Study With Young People
Source: JMIR Form Res. 2024 Apr 23;8:e50368. doi: 10.2196/50368 (PMC11077411; doi:10.2196/50368)
Supplement: Multimedia Appendix 2 [file formative_v8i1e50368_app2.docx]

| Question | Possible options |
| --- | --- |
| Who can access the data? | Anyone  People with certain jobs  People with certain skills  People in certain places |
| Where is the data hosted? | One place  Many places |
| Who controls the data? | No one  Community decides  Community review panel  Community hires manager |
| What do people have to do before they can access the data? | Ethics training  Provide ID  Review board approval  Sign contract  Pay money |
| Who takes on the cost of managing the data? | People who access it  Government  Organisation/institution  Private company |
| How can people see the data? | Download it  View it in a server  View a recreated dataset |
| What kind of research can people do on the data? | Anything  Certain types of analysis  Certain types of project |

Seven key questions asked in deliberative democracy sessions and suggested responses
